# Electrostatic Interactions of Fluorescent Molecules with Dielectric Interfaces Studied by Total Internal Reflection Fluorescence Correlation Spectroscopy

**DOI:** 10.3390/ijms11020386

**Published:** 2010-01-28

**Authors:** Hans Blom, Kai Hassler, Andriy Chmyrov, Jerker Widengren

**Affiliations:** Department of Biomolecular Physics, Royal Institute of Technology, Stockholm, Sweden

**Keywords:** electrostatic interactions, fluorescent molecules, glass interfaces, Total Internal Reflection, Fluorescence Correlation Spectroscopy

## Abstract

Electrostatic interactions between dielectric surfaces and different fluorophores used in ultrasensitive fluorescence microscopy are investigated using objective-based Total Internal Reflection Fluorescence Correlation Spectroscopy (TIR-FCS). The interfacial dynamics of cationic rhodamine 123 and rhodamine 6G, anionic/dianionic fluorescein, zwitterionic rhodamine 110 and neutral ATTO 488 are monitored at various ionic strengths at physiological pH. As analyzed by means of the amplitude and time-evolution of the autocorrelation function, the fluorescent molecules experience electrostatic attraction or repulsion at the glass surface depending on their charges. Influences of the electrostatic interactions are also monitored through the triplet-state population and triplet relaxation time, including the amount of detected fluorescence or the count-rate-per-molecule parameter. These TIR-FCS results provide an increased understanding of how fluorophores are influenced by the microenvironment of a glass surface, and show a promising approach for characterizing electrostatic interactions at interfaces.

## 1. Introduction

Investigations of electrostatic interactions, taking place on or in the vicinity of surfaces, are important for gaining a deeper understanding of interfacial dynamics. For example, enzymology studies have elucidated how enzymes can make beneficial use of a strong attractive electric field (produced by charges on the enzyme surface) to enhance enzyme-substrate association [1,2]. Differences in transport selectivity of ions through membrane-spanning proteins have also been attributed to distinct electrostatic properties of the funneled proteins [3]. These examples resemble, to a large extent, how transport of charged particles (electrons and holes) is controlled in modern computer chips by intrinsic doping of charge densities and externally applied electric fields [4]. Interfacial electrostatic interactions also play a key role during DNA transcription and replication. Recent findings show that week repulsion allows sliding of DNA binding proteins along the strain until attraction may occur at specific recognition sequences [5]. Electrostatic interactions at interfaces also play a key role in the field of separation science, *i.e.*, chromatography or capillary electrophoresis [6]; where the separation dynamics depend upon the analyte components, the mobile solution phase, and the surface properties of the packing or capillary support material. Given the key role of separation techniques for biomedical, pharmaceutical, and environmental analyses a large amount of techniques have been developed to characterize interfacial dynamics [6].

Total Internal Reflection Fluorescence Microscopy (TIR-FM) is a suitable technique to study the mobility of fluorescent particles or molecules near surfaces. By exciting with evanescent laser excitation a very thin layer at the surface (typically about one hundred nanometers thick), one elegantly confines the probed volume and allows interfacial dynamic investigations [7]. Using TIR-FM, the restricted motion of single organic dye molecules has been observed at fused-silica surfaces [8]. The electrostatic contribution to adsorption on such surfaces has also been studied [9]. TIR-FM has further been applied to investigate the restricted motion of single dye-labelled protein molecules and single intercalator-labelled DNA molecules at fused-silica surfaces at various pH and ionic strengths [10,11]. The technique has additionally been used to investigate the dynamic properties of fluorescent beads in the vicinity of bare and coated glass surfaces [12–14].

The motion of fluorescent probes at interfaces can further be analyzed by Fluorescence Correlation Spectroscopy (FCS) [15]. The principle behind this analysis is the detection of fluorescence fluctuations from a small probe volume followed by statistical evaluations of the time-dependence of these fluctuations, brought about by the dynamics of individual fluorescent molecules [16]. In its simplest form, the fluctuations are governed by molecules diffusing in and out of the probed volume [17]. Directed movements, such as flow in and out of the probe volume, may also contribute to the fluorescence fluctuations [18–20]. Furthermore, chemical reaction kinetics, conformational changes, protonation reactions or photophysical processes, may also cause the fluorescence to fluctuate [16,17,21–23]. By use of FCS, it is in principle possible to deduce information about any dynamical process that manifests itself as a change in fluorescence intensity. To obtain the information, the fluorescence signal is analyzed in terms of correlation functions that give qualitative information of dynamic entities, such as, diffusion coefficients, chemical reaction rates, flow speeds, triplet-state kinetic rates, *etc*. [24,25].

By combining FCS with evanescent laser excitation, known as Total Internal Reflection Fluorescence Correlation Spectroscopy (TIR-FCS), restricted motion of fluorescently labelled antibodies near phospholipid bilayers have been studied [26,27]. The restricted motion of fluorescently labelled vesicles diffusing near lipid membranes has also been investigated with TIR-FCS [28]. The technique has additionally been used to investigate electrostatic contributions to adsorption kinetics of organic dye molecules in the vicinity of bare and coated silica surfaces [29]. Evanescent laser excitation and fluorescence detection is either done through the same objective [30], which is different to the more common system that uses prism-based total internal reflection excitation, and an additional objective for fluorescence detection [15]. With the former method we have investigated diffusion of dianionic and cationic organic dyes molecules in pure aqueous solution at glass interfaces [31,32]. In addition, we have further applied it for investigation of DNA-hybridization, single enzymes kinetics, triplet-state dynamics, and protein and surfactant interactions at bare and coated glass surfaces [33–36].

In this work, objective-based TIR-FCS is applied to investigate electrostatic interaction of fluorescent molecules with different charge (cationic, zwitterionic/neutral, and anionic/dianionic) and negatively charged dielectric glass surfaces, under different ionic strengths around physiological pH. The large information content available with TIR-FCS is used to deduce concentration changes, mobility variations, and photophysical kinetics parameter. As analyzed by means of the amplitude and time-evolution of the autocorrelation function, the fluorescent molecules experience electrostatic attraction or repulsion at the glass surface depending on their charges. Influences of the electrostatic interactions are also monitored through the triplet-state population and triplet relaxation time, including the amount of detected fluorescence or the count-rate-per-molecule parameter.

## 2. Experimental Section

### 2.1. Evanescent Excitation

The physical aspects of evanescent excitation using TIR have been described previously [7]. As a laser beam propagates through a high refractive index material (glass *n*_1_ = 1.52) and encounters a low refractive index material (water *n*_2_ = 1.33), usually a small portion is reflected and the majority is transmitted while being refracted towards the interface. For total internal reflection to occur, the sine of the angle of incidence (measured from the normal of the interface) must exceed the ratio of the refractive indices of the interfacing media, *i.e.*,*θ*> sin^−1^(*n*_2_/*n*_1_) ≈ 61°. At and above this critical angle the propagating beam is totally back-reflected from the interface. However, in the vicinity of the interface there is a non-propagating evanescent field. This field is used to excite fluorescent molecules in all TIR applications [15]. In objective-based TIR, an excitation field with an irradiance that is approximately Gaussian in the lateral direction, *ρ*, and exponential in the axial direction, *z*, is generated [32]:

(1) $$I(\text{r})=\eta \frac{2P}{\pi R^{2}}\text{exp}(-2\rho^{2}/R^{2})\text{exp}(-z/d)$$

**Figure 1.**
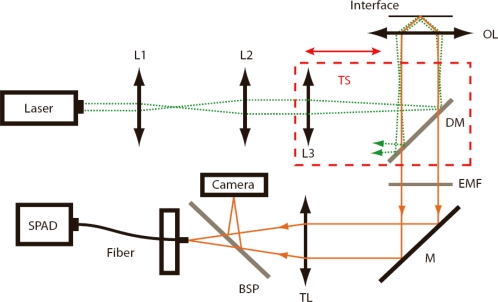
Schematic of the objective-based TIR-FCS setup. L1–L3: lenses; TS: translator; DM: dichroic mirror; OL: objective lens; EMF: emission filter; M: mirror; TL: tube lens; BSP: beamsplitter plate; SPAD: single photon avalanche diode.

The characteristic axial decay length at which this irradiance decays by a factor of 1/*e* is given by the penetration depth, $d=\lambda_{0}/4\pi {\left(n_{1}^{2}{\text{sin}}^{2}\theta -n_{2}^{2}\right)}^{-1/2}$, where *λ*_0_ is the excitation wavelength in vacuum. The parameter *P* is here the incident laser power and *R* is the lateral radius of the excitation area at the interface. The level of irradiance experienced by a fluorescent molecule at the surface may be greater than the irradiance from the incident laser beam by up to a factor of five [7]. Under our experimental conditions the angle of incidence (AOI) is fixed to a value slightly below 64° (estimated as 63.7°), which generates an enhancement factor of *η*~4 (see reference [36] for a discussion of AOI settings). The discontinuity in the refractive index also produces a significant modification of the angular dependence of the emitted fluorescence, which has a maximum of emission in the direction of the critical angle (*i.e.*, towards the high index material) [37]. A fraction that is significantly higher than 60% of the total emitted power is radiated into the medium with the high refractive index and most of this power is detected in an objective-based system [30]. In prism-based system the fraction of detected power is comparatively low [15]. This results in increased fluorescence signals detected by objective-based TIR systems [31,32] (see Figure 1 for setup).

### 2.2. Correlation Analysis

In fluorescence correlation spectroscopy (FCS), the fluctuating fluorescence, *F*(*t*), is analyzed by means of its autocorrelation function, *G*(τ), which can be defined as:

(2) $$G(\tau )=\langle F(t)F(t+\tau )\rangle /{\langle F\rangle }^{2}-1$$

where brackets denote averaging over time, *t*. The detected fluorescence is further assumed to be proportional to the time-dependent concentration, *C*(*r*,*t*), and the molecule detection efficiency function, MDF(**r**), which may be expressed as [24]:

(3) F(t)=β∫MDF(r)C(r,t)dV

Here, the MDF function, sometimes called the apparent excitation profile, determines the detection volume and is defined as the TIR excitation profile multiplied with the collection efficiency function of the microscope [15,32]. The parameter *β* accounts for the quantum efficiency of the detectors as well as the geometrical and optical filtering losses inherent in the experimental arrangement, including the fluorescence quantum yield of the fluorescent molecules. Assuming the detected fluorescence to be given by Equation (3), the following analytical correlation function can be deduced [32]:

(4) $$G(\tau )=\frac{\gamma }{\bar{N}}\left[\left(1-\frac{\tau }{2\tau_{z}}\right)w\left(i\sqrt{\frac{\tau }{4\tau_{z}}}\right)+\sqrt{\frac{4\tau }{\pi \tau_{z}}}\right]{\left(1+s^{2}\frac{\tau }{\tau_{z}}\right)}^{-1}\left\{1+\frac{\bar{T}}{1-\bar{T}}\text{exp}\left(-\frac{\tau }{{\bar{\tau }}_{T}}\right)\right\}$$

In this Equation, which describes the time-dependence of free diffusion and triplet-state kinetics, *N̄* is the mean number of molecules within the probed volume. Stated differently, the limit of *G*(τ) as *τ* goes to zero is given by the inverse number of molecules in the volume. The average axial diffusion time is defined as *τ_Z_* = h^2^/4D, where *D* is the diffusion coefficient and *h* is the axial extent of the volume, which is approximately equal to the penetration depth. Expression *w* is the complex generalization of the error function defined as *w*(*x*) ≡ exp(−*x*^2^)*erfc*(−*ix*) and *γ* ≡ ∫MDF^2^ (**r**)*dV/*∫MDF(**r**)*dV* is the geometrical correction factor for the volume. The latter was derived through numerical calculations and set to 0.294 in this work [32]. The parameter *s* = *h*/*b* describes a structure factor for the axial-to-radial dimension of the detection volume, with *b* being the radius in the lateral direction (1/*e*^2^ value) defined by the size of the detector aperture. The structure factor was in this work fixed to 0.18 [32]. The parameter, *T̄* is the steady state probability of the molecules to be in the triplet state, and *τ̄_T_* denotes the steady state triplet relaxation time [36]. To fit the TIR-FCS data, a multidimensional least-squares minimization algorithm written in Matlab (Mathworks Inc., Natick, MA, USA) was used to estimate the unknown parameters of the Equation above, *i.e.*, *N*, *τ_Z_*, *T̄*, and *τ̄_T_*, where presented values are averages of a set of three 60 seconds measurements.

### 2.3. Electrostatic Interactions

**Figure 2.**
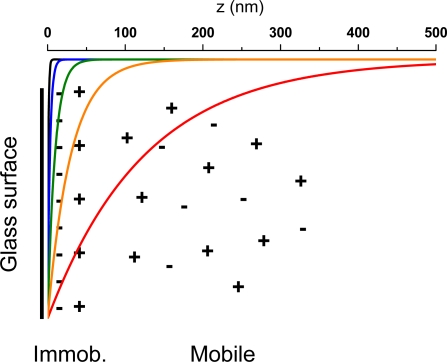
Schematic model of electric double layer. The negative surface charges (−) are counterbalanced by bound and free ions (+). The electrostatic surface potential is plotted for salt concentrations of 100 mM, 10 mM, 1 mM, and 0.1 mM, which gives Debye lengths of 0.95 nm (black curve), 3 nm (blue curve), 9.5 nm (green curve), and 30 nm (orange curve), respectively. The evanescent penetration depth is for comparison plotted in the red curve.

When a solid material is in contact with an aqueous solution, a thin interfacial charge layer is formed, which consist of surface charges and charge balancing counterions [38]. The presence of the surface charges causes a rearrangement of the ions in the vicinity of the surface. This arrangement of a nonzero net charge at the solid-liquid interface is usually dubbed the electric double layer. In the electric double layer, two regions of charge distributions may be identified. Immediately next to the charged surface, counterions are bound to the surface due to strong electrostatic attraction. Outside of this immobile layer counterions may move, meaning that they can diffuse in the potential set up by the partially screened surface. The immobile region is often referred to as the compact layer or the Stern layer, and the mobile region is dubbed the diffusive layer. The electrostatic potential at the boundary dividing the compact layer and the diffusive layer is the so-called zeta (ζ) potential [6]. A schematic model of the electric double layer on a glass surface is shown in Figure 2. On uncoated glass surface, acidic silanol groups acquire a negative charge (SiO^−^) at neutral pH. This negative surface charge density is balanced by counterions attracted to the surface and diffusive ions counterbalancing the potential set up by the partially screened surface. On uncoated hydrophilic surfaces such as glass, the surface potential has been shown to be indistinguishable from the zeta potential [13].

How rapid the surface potential drops is connected to the Debye-Hückel parameter, which for dilute electrolytes can be expressed as *κ*^2^ = 2*χe*^2^ / *ɛ_r_ɛ*_0_*k_B_T* [38]. Here *χ* is the ionic strength of the solution, *e* is the elementary charge and the parameters *ɛ_r_* and *ɛ*_0_ are the permittivity of the electrolyte and that of vacuum, *k_B_* is the Boltzmann constant and *T* is the absolute temperature. The reciprocal of *κ* correspond to the fundamental length scale of the surface potential, or in other words to the thickness of the electric double layer. This so-called Debye screening length, *z_D_*, depends on the ionic strength of the solution, *χ*, and can at room temperature be expressed as $\kappa^{-1}=z_{D}=0.3/\sqrt{\chi }$ in units of nanometer [38]. At an ionic strength of 150 mM, the Debye length is below 1 nm (0.77 nm) and concentration changes at the glass surface governed by electrostatic interactions are here assumed negligible (*i.e*., basically a neutral surface for diffusive molecules as the molecular size of the organic dye molecules are around 1 nm) [29]. At ionic strengths between 100 mM and 0.1 mM, the *z_D_* values range from 0.95 nm to 30 nm. In comparison, the penetration depth is around 125 nm for the conditions used in this work. Figure 2 shows the surface potential at the different ionic strengths, together with the evanescent excitation profile given by the *z*-dependent part of Equation (1). The electrostatic surface potentials are plotted according to Gouy-Chapman theory that assumes it to decay exponentially with distance to the interface: Ψ(*z*) = Ψ_0_ exp(−*z* / *z_D_*) [38].

Assuming a Poisson-Boltzmann description for the number of fluorophores (at thermal equilibrium) in the vicinity of the surface [13,38], the one-dimensional concentration distribution may be approximated as:

(5) $$\bar{C}(\text{r})=\bar{C}(z)={\bar{C}}_{B}\times \text{exp}(-qe\Psi (z)/k_{B}T)$$

Here *q* is the charge value of the fluorescent molecule probing the screened glass surface (*q* = +1, ±0, −1, −2) and *C̄_B_* is the concentration in the bulk (*i.e.*, far away from the surface). In TIR-FCS, the number of molecules is determined exclusively by the concentration profile, *C̄*(*z*), and the dimension and shape of the detection volume. While there are no (well-defined) boarders of the detection volume, its absolute size is exactly defined and corresponds to the volume containing *N̄* molecules as defined *via* Equation (4), *i.e.*, *C̄*(*z*) ∝ *N̄* = *γ*[*G*(0)×(1−*T*)]^−1^. Similarly, the bulk values *N̄_B_* ∝ *C̄_B_* can be approximated with the number of molecules found in the volume at a “fully” screened surface. At negatively charged glass surfaces, depending on the charge of the fluorescent molecule, three cases of concentration variations can thus be identified. No concentration changes at all (*i.e.*, *N̄* equal to *N̄_B_*) should be generated when *q* is zero. Fluorescent molecules having a positive *q*-value should be attracted towards the surface (*N̄* larger than *N̄_B_*), whereas a negative *q*-value would lead to repelling of molecules by the surface (*N̄* smaller than *N̄_B_*). By measuring the excess (or deficit) of molecules with TIR-FCS, a mean surface potential may thus be evaluated for each ionic strength *via* Equation (5). The errors in our FCS and surface potential values are estimated to be about 5% and 10%, respectively.

The autocorrelation’s amplitude (and time-dependence) is in Equation (4) derived assuming free diffusion. To extend this analysis, a direct numerical modeling of the correlation amplitude affected by the electrostatic contribution is also performed. As the correlation function depends on the concentration profile, the amplitude of the former can be expressed as:

(6) $$G(0)=\frac{1}{{\bar{C}}_{B}}\frac{\int {\text{MDF}}^{2}(\text{r})\text{exp}\left[-\frac{qe\Psi_{0}}{k_{B}T}\text{exp}(-z/z_{D})\right]dV}{{\left\{\int \text{MDF}(\text{r})\text{exp}\left[-\frac{qe\Psi_{0}}{k_{B}T}\text{exp}(-z/z_{D})\right]dV\right\}}^{2}}$$

where Equation (3) has been inserted into Equation (2) and the electrostatic potential is approximated by Gouy-Chapman theory [38]. Equation (6) then expresses the correlation amplitude as the detection profile multiplied with the electrostatic concentration profile decay. This expression can further be rewritten and simplified using $V_{eff}={\left\{\int \text{MDF}(\text{r})dV\right\}}^{2}/\int {\text{MDF}}^{2}(\text{r})dV$ and $a=-\frac{qe\Psi_{0}}{k_{B}T}$ to give:

(7) $$\frac{2h}{V_{eff}{\bar{C}}_{B}}\frac{\int \text{exp}(-2z/h)\text{exp}[a\;\text{exp}(-z/z_{D})]dz}{{\left\{\int \text{exp}(-z/h)\text{exp}[a\;\text{exp}(-z/z_{D})]dz\right\}}^{2}}-G(0)=0$$

Expression (7) for the distribution of fluorescent molecules in the detection volume may then, for each ionic strength (*i.e.,* $z_{D}=0.3/\sqrt{\chi }$), be solved numerically for the parameter *a*, allowing the surface potential to be extracted from the measured correlation amplitude, *G*(0). It should be noted that at short Debye lengths (*i.e.*, high ionic strengths), the argument of the concentration profile part above becomes very large, making expression (7) very sensitive to accurate *z_D_* values.

### 2.4. Objective-based TIR-FCS Setup

Figure 1 schematically shows our objective-based TIR-FCS setup, which uses a single line 491 nm diode laser (Calypso, Cobolt AB, Stockholm, Sweden), which power is controlled by a variable neutral density filter (NDC-25C-4 Thorlabs Inc. Newton, NJ, USA). An achromatic lens with focal length of 200 mm focuses the 3.5 times expanded laser beam onto the back-focal plane of an oil immersion objective (α-Plan-Fluar 100× NA 1.45, Carl Zeiss, Jena. Germany). The laser beam is reflected into the objective using a dichroic mirror (F500-Di01, Semrock Inc. Rochester, NY, USA). A beam offset of about 2.2 mm from the optical axis results in evanescent field excitation, with a lateral diameter at the glass/water interface of about 20 μm (1/*e*^2^-value), as measured by an electron-multiplying CCD camera (iXon DU-860, Andor Technology, Belfast, N. Ireland). The resulting irradiance used in the present work ranged from 2 – 10 kW/cm^2^. The focusing lens and the dichroic mirror are moved in one block by a linear translator with micrometer screws to adjust the lateral position of the laser beam entering the objective. In this way, the excitation angle can be adjusted without altering the optical path length between the focusing lens and the objective. To keep the focusing position to the glass-water interface a closed-loop piezo stage with 5 nm resolution (Nanomax TS, Thorlabs) is used. The emitted fluorescence is collected with the same high NA objective and focused with an achromatic tube lens onto the core of a 50 μm multimode fiber (Fibertech, Berlin, Germany), which is connected to a single photon avalanche photodiode (SPAD-AQR-14-FC, PerkinElmer Optoelectronics, Salem, MA, USA). A beamsplitter plate (G344132000, Linos, Göttingen, Germany) is used to split the fluorescence between the detector and the camera. Band-pass filters (HQ 550/80, Chroma, Bellows Falls, VT, USA) are placed in front of the detector and camera to block back-reflected and scattered laser light. The detected signal is finally autocorrelated online using an ALV-6000 correlator (ALV Erlangen, Germany).

### 2.5. Organic Dyes

Fluorescein, rhodamine 110 (Rh110) and rhodamine 123 (Rh123) are purchased from Molecular Probes (Carlsbad, CA, USA) and rhodamine 6G perchlorate (Rh6G) and ATTO 488 are purchased from Atto-Tec GmBH (Siegen, Germany). The molecular masses of the dyes are: fluorescein—389 g/mol, rhodamine—110–367 g/mol, rhodamine—123–381 g/mol, rhodamine 6G—479 g/mol, and ATTO—488–591 g/mol. Stock solutions of the different dyes are prepared in spectroscopically pure ethanol or dimethylsulfoxide (DMSO) and these are prior to measurements diluted in ultrapure water to nanomolar concentrations and different ionic strengths (addition of analytical grade sodium chloride). The buffer used in some of the TIR-FCS experiments is PBS (pH = 7.2) with and without added sodium chloride. All samples are mixed prior to pipetting them onto the clean glass surfaces and the accuracy of the final sample concentration in estimated to be about 5% of the stated values.

### 2.6. Surface Preparations

The surface preparation of cleaned glass microscope cover slips (No. 1, Ø = 25 mm Hecht-Assistent, Sondheim, Germany) is done according to the following protocol: cleaning for 20 min. in an ultrasonic bath in a solution of 2% Hellmanex II (Hellma, Müllheim, Germany) and thereafter thorough rinsing in ultrapure water from a Barnstead EASY pure purification system; ultrasonic cleaning for 20 min. in an acetone/ethanol mixture (30:70% by volume) and thorough rinsing; ultrasonic cleaning for 20 min. in water and rinsing with water and spectroscopically pure ethanol. The cover glasses are blown dry with nitrogen and oxygen plasma etched for two minutes in a reactive ion etcher (PlasmaLab 80 Plus, Oxford Instrument, Oxford, UK). The cover glasses are finally rinsed with ultrapure water and blown dry with nitrogen prior to measurement. An elastic Teflon ring with inner diameter Ø = 8 mm is placed on top of the clean hydrophilic cover glasses to prevent the samples from spreading into a thin film. This configuration generates a sample reservoir in the form of a standing droplet, which in addition prevents fast evaporation. All TIR-FCS measurements were then initiated after a 60 seconds equilibration time.

## 3. Results and Discussion

**Figure 3.**
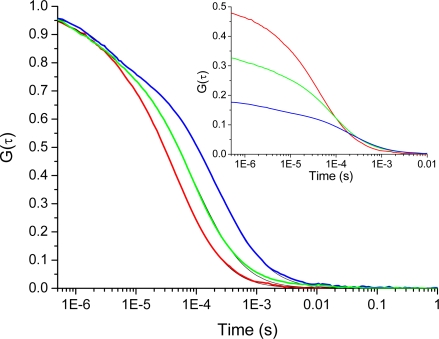
Autocorrelation curves for the cationic rhodamine 123 at ionic strengths of 100 mM (red curve), 10 mM (green curve) and 0.1 mM (blue curve). The correlation curves are fitted with Equation (4) to estimate the unknown parameters *N̄*,*τ_z_*, *T̄*, and *τ̄_t_*. To visualize the effect of the electrostatic interactions on the diffusion time, the curves have been normalized. The inset shows the unnormalized curves, which are used to visualize changes in concentration of molecules in the vicinity of the glass interface. At ionic strength of 100 mM: *N̄* = 0.62, *τ_z_* = 4.9 μs, *T̄* = 0.057 and *τ̄_t_* = 1.6 μs; at 10 mM: *N̄* = 0.96, *τ_z_* = 9.7 μs, *T̄* = 0.1 and *τ̄_t_* = 2.1 μs; at 0.1 mM: *N̄* = 1.93, *τ_z_* = 23.5 μs, *T̄* = 0.17 and *τ̄_t_* = 3.1 μs. Concentration of Rh123 was 20 nM.

**Figure 4.**
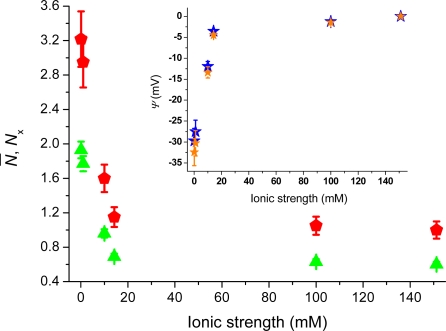
Mean number of rhodamine 123 molecules in the TIR-FCS probe volume, *N̄* (red pentagon), and the ratio of molecules, *N*_x_ (green triangles), as a function of ionic strength. The inset shows the deduced mean surface potential of the electric double layer without (blue stars) and with (orange stars) correction for background contributions. Data points at 14 mM and 150 mM is done in PBS at pH = 7.2. Data points at 0.1 mM, 1 mM, 10 mM, and 100 mM are done at pH = 6.6.

Figure 3 shows the TIR-FCS autocorrelation curves for cationic rhodamine 123 interacting with the negative glass surfaces at different ionic strengths. The influence of surface potential on the molecular motion manifests itself in an increased decay-time with decreasing ionic strength (*i.e.*, a less screened surface potential). The inset shows how the concentration of molecules, in the vicinity of the surfaces, increases as the ionic strength decreases from 100 mM to 0.1 mM. This is reflected in a decrease in the amplitude of the correlation curve. Both the shift in amplitude, as wells as the shift in decay-time of the correlation curves, clearly shows that the positively charged rhodamine 123 is attracted to the negatively charged dielectric surface. The small deviations seen at long times are most probably due to rare adsorption/desorption events not included in the present analysis. Figure 4 shows the number of molecules, *N̄*, deduced from the correlation amplitudes together with the ratio of molecules, *N*_x_ = *N̄* / *N̄_B_*, used *via* Equation (5) to evaluate the surface potential plotted in the inset, *i.e.*, Ψ(z) = Ψ = ζ. For an ionic strength of 0.1 mM, we deduce ζ~–30 ± 3 mV (pH = 6.6), with the bulk concentration, *N̄_B_*, estimated from the data at 150 mM. The strength of the mean potential is slightly lower than values reported previously for glass surfaces. Investigations with TIR-FM on glass coverslips probed by fluorescent nanoparticles gave ζ~–44.4 mV (pH = 6.5) [12]. TIR-FM studies on Pyrex glass gave ζ~–66 ± 8 mV (unknown pH) [13]. For polished cover slips a value of ζ~–87 mV (pH = 6.5) was measured by electro-osmosis [39]. As the literature values show, measured surface potentials depend on the kind of glasses used and on differences in preparation and cleaning protocols (wet and dry steps). The applied techniques also evaluate somewhat different parameters.

In electro-osmosis, the velocity of a liquid in response to an applied electric field in, for example, a capillary is used to measure the surface potential [6]. In TIR-FM, the detected fluorescence intensity is used to estimate the position of fluorescent particles at the surface as a function of *z*-position. In principle, this information is encoded in the detected fluorescence *via* the exponential dependence of the evanescent excitation, *i.e*., Equation (1), which magnifies small axial position changes into large fluorescence changes. However, as the fluorescence emission is highly non-isotropic at an interface [37], the correlation between distances and detected fluorescence measurements can be biased [12].

In TIR-FCS, the number of molecules at the interface is extracted through the amplitude of the correlation curve. The amplitude can likewise be biased by contributions of uncorrelated background signals, consisting for example of back-reflected and scattered laser light. Contribution of such background signals decreases the amplitude of the correlation curve and leads to an overestimation of the number of molecules in the probed volume, *i.e.*, *N̄*. To correct for bias in the amplitude, the “true” number of molecules, *N*_0_, may be extracted using the following expression: *N̄*_0_ = *N̄* (1 + *B*/*F*)^−2^, where *B* is the background signal that can be measured for a sample of pure water, and *F* is the detected fluorescence signal [24,40]. Incorporating this correction, with a *B*/*F* ratio that varied from 0.07 to 0.02 as the ionic strength increased from 0.1 mM to 150 mM, leads to a modest change of the surface potential from −29.7 mV to −32.4 mV at 0.1 mM (cf. blue and orange stars in the inset of Figure 4). Note that when error-bars are not visible, the errors are contained in the size of the markers.

**Figure 5.**
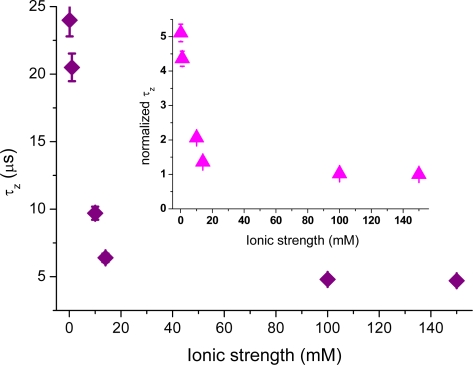
The axial passage-time, *τ_z_*, for rhodamine 123, as function of ionic strength (purple squares), deduced by fitting the autocorrelation data with Equation (2). The inset shows the normalized axial passage-time (pink triangles).

This unbiased value can further be compared to the numerical analysis of expression (7), which incorporates the effect of the electrostatic concentration profile to the correlation amplitude. Applying this expression to the measured values of the amplitude, *i.e.*, *G*(0) = *γ*[*N̄*×(1–*T*)]^−1^, yields a surface potential of about −94 ± 8 mV at an ionic strength of 0.1 mM, which is higher than that deduced directly *via* Equation (5). The bulk concentration *N̄_B_* = *V_eff_* *C̄_B_* was extracted at 150 mM ionic strength, and the axial extent of the detection volume, *h*, was deduced by the axial passage time *τ_z_*, *via* $h=\sqrt{4D\tau_{z}}$, assuming D~4.1 × 10^−10^ m^2^s^−1^ [41] (cf. Figure 5). The numerically extracted surface potential is slightly higher than the cited literature values [12,13], but agrees fairly well with values for mechanically polished glass [39]. This might be explained as an effect of our oxygen plasma etching step, which probably increases the density of attracting silanol groups on the glass surface.

Furthermore, the attraction of cationic rhodamine 123 towards the negative glass surface manifests itself in a change in the main decay-time of the correlation curves (cf. Figure 3). Figure 5 shows the dependence of the average axial passage-time, *τ_z_*, on the ionic strength, deduced by fitting the autocorrelation data with Equation (4). A large increase in the axial passage-time through the probe volume located at the glass surface is observed with decreasing ionic strength. The same increase as seen at 0.1 mM ionic strength was also seen in (pure) aqueous solution having pH = 6.6 (data not shown). By normalizing the axial passage-time, shown in Figure 5, with the passage time given for a fully screened surface a plot resembling that of the increased concentration profile, *N*_x_, shown in Figure 4 is generated. The inset shows that the normalized passage time of the cationic molecules can be prolonged up to a factor of five, meaning that they spend a considerably longer time in the probed volume at low ionic strengths. From the data shown in Figure 5 it should therefore in principle be possible to deduce the surface potential, through a model of charged particle diffusing in an electric potential as introduced by Smoluchowski [42]. However, the nonlinearity of the potential makes it difficult to derivate an analytical expression for the autocorrelation function. Fitting of the electrostatic affected axial passage time is hence performed (so far) with an expression assuming free diffusion, *i.e.*, Equation (4). In previous studies, the diffusion coefficient of anionic antibodies at cationic surface membranes was seen to be larger at low ionic strengths and smaller at high ionic strengths [26], which is opposite to what is seen in Figure 5. Note that the inverse of the axial passage-time is proportional to the diffusion coefficient (*i.e.*, *τ_z_* ∝ 1/*D*). This opposite relation could be explained by influences from hydrodynamic interaction between the antibody and the membrane surface [27]. Interfacial interaction on coated surfaces, is therefore often a somewhat more complex dynamic process, and the use of different coatings may actually amplify both hydrophobic [13,29], as well electrostatic interactions [13,39]. Also the hydrophobicity of the probe molecules may influence the surface dynamics [29], which leads to chemical interaction occurring simultaneously with charge driven electrostatic contributions.

**Figure 6.**
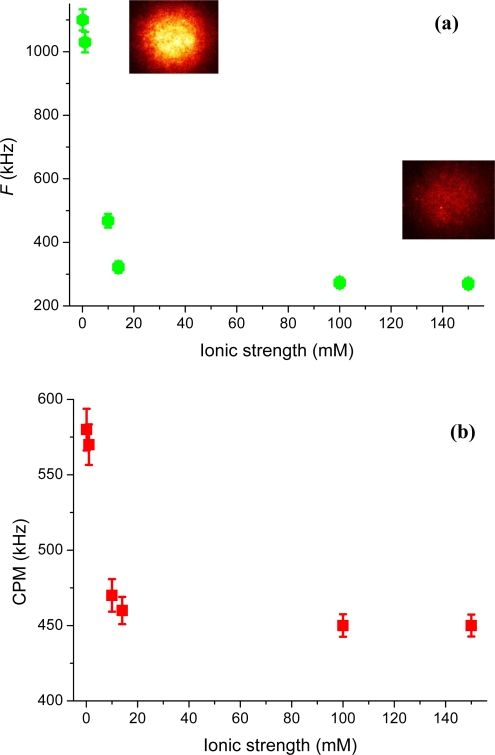
(a) The SPAD detected fluorescence, *F*, as function of ionic strength (green hexagons). The inset shows the detected CCD intensities at 0.1 mM (left) and 150 mM (right) ionic strength. The camera images are presented with the same peak intensity setting and the diameter correspond to 20 μm in sample space. (b) The counts-rate-per-molecule, CPM, as a function of ionic strength (red squares).

The electrostatic interaction at our uncoated glass surface can also be investigated through the detected fluorescence on the SPAD detector, or on the CCD camera. Figure 6a shows the detected fluorescence, *F*, as a function of ionic strength. Images of the detected fluorescence for rhodamine 123, monitored on the CCD at 0.1 mM and 150 mM ionic strength, are also shown. The deduced count-rate-per-molecule (in kHz), calculated as CPM = *F*/ *N̄*, is further shown in Figure 6b. A comparison between the detected fluorescence and the CPM reveals that the former increases by a factor of four, where as the latter only increases about 1.3 times when the ionic strength is lowered. This is understandable as more (attracted) cationic molecules contribute to the signal at low ionic strength and are adding further to the detected signal through the larger (on average) experienced excitation irradiance (*i.e.*, being closer to the surface). However, the added signal does not increase linear with the excitation irradiance as saturation of the fluorescence emission may occur.

Emission rates of fluorescent molecules are largely determined by the excitation irradiance (*i.e.*, W/cm^2^) at which saturation of the singlet-state is reached, and this in turn depends on the mean occupancy of the long-lived triplet state [23]. While the fluorescence lifetime of the singlet-state of an organic dye molecule typically is in the nanosecond range, the lifetimes of the triplet state is in the microsecond to millisecond range, depending on the environment of the molecule. Consequently, the triplet state has ~10^3^–10^6^ more time to interact with the immediate environment, compared to the fluorescent singlet state. The kinetics of the triplet state thus represents an additional dimension of information, which can respond and change considerably due to small changes in the microenvironment, e.g., accessibility of quencher molecules or solvent viscosity [23].

**Figure 7.**
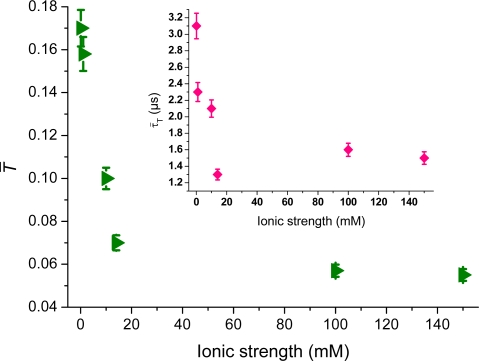
The triplet amplitude, *T̄*, describing the probability of the rhodamine 123 molecules to be in the triplet state, as function of ionic strength (green triangles). The inset shows the triplet relaxation time, *τ̄_T_* (pink squares).

In this work, the influence of the presence of the glass surface on the triplet-state dynamics is also studied. Figure 7, shows the changes of the TIR-FCS deduced triplet amplitude, *T̄*, as function of the ionic strength for rhodamine 123. This is deduced by fitting Equation (4) to the autocorrelation data shown in Figure 3. When decreasing the ionic strength, the triplet-state population is increased in response to the increased electrostatic attraction, shifting more cationic molecules towards the larger excitation irradiances at the surface. The inset shows the triplet relaxation time, *τ̄_T_*, that shows a similar trend as the triplet-amplitude. This is in agreement with the population kinetics of the triplet state, where longer *τ̄_T_* values generate larger *T̄* values. Triplet-state kinetics of organic dye molecules in the vicinity of glass surfaces has been investigated previously at high ionic strengths (*i.e.*, fully screened surfaces) [36]. Extracting the triplet-state kinetic rates from the data on unscreened surfaces should in principle be possible, but is beyond the scope of this work. The non-isotropic concentration variation, due to the electrostatic concentration profile, then needs to be included in that rate analysis.

**Figure 8.**
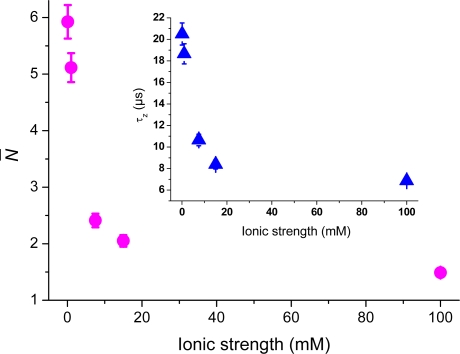
The number of rhodamine 6G molecules, *N̄* (pink circles) in the TIR-FCS probe volume, as a function of ionic strength. The sample concentration was 50 nM and the values have been corrected for background contributions. The inset shows the axial passage-time, *τ_z_* (blue triangles).

**Figure 9.**
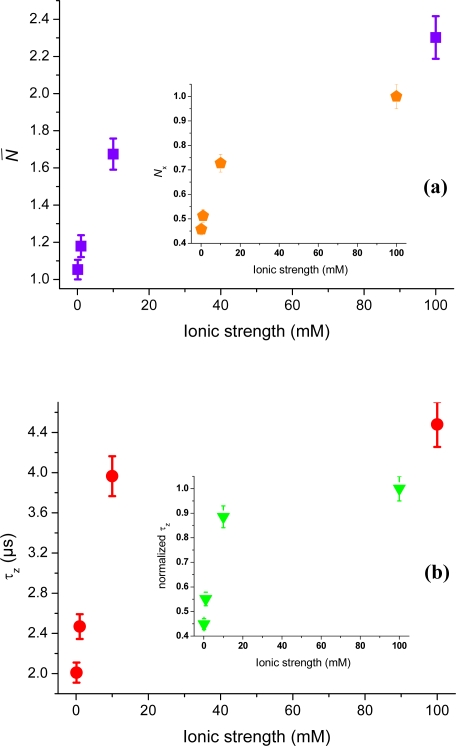
(a) The number of fluorescein molecules, *N̄* (purple squares) in the TIR-FCS probe volume, as a function of ionic strength. The concentration variation ratio of molecules, *N*_x_, is shown in the inset (orange pentagons). The sample concentration was 100 nM. (b) The axial passage-time, *τ_z_*, for fluorescein as function of ionic strength (red circles). The inset shows the normalized axial passage-time (triangles).

**Figure 10.**
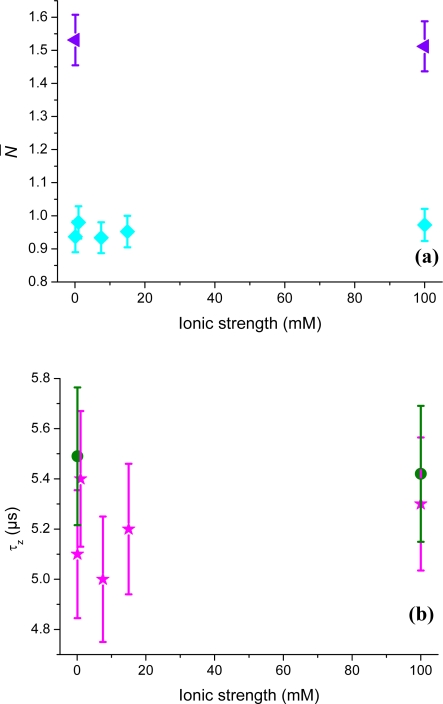
(a) The number of rhodamine 110 (purple triangles) and ATTO 488 (cyan squares) molecules, *N̄*, in the TIR-FCS probe volume, as a function of ionic strength. The sample concentration of rhodamine 110 and ATTO 488 were 50 nM and 40 nM, respectively. (b) The axial passage-time, *τ_z_*, of rhodamine 110 (green hexagons) and ATTO 488 (pink stars).

The examples above show the potential TIR-FCS has for investigating electrostatic interaction dynamics of single fluorescent molecules at interfaces. Figures 8–10 show additional examples of this for cationic rhodamine 6G, anionic/dianionic fluorescein, zwitterionic rhodamine 110, and neutral ATTO 488, monitored at various ionic strengths at negatively charged glass surfaces. As can be seen in Figure 8, cationic rhodamine 6G shows a similar increase in number of molecules as cationic rhodamine 123 (cf. Figure 4). The values are corrected for background contributions and all data points are obtained for molecules in aqueous solution at ionic strengths between 0.1 mM and 100 mM (pH = 6.6). Assuming that the surplus of rhodamine 6G molecules at the interface is due to electrostatic attraction, Equation (5) yields a surface potential of about −35 ± 4 mV (pH = 6.6) at 0.1 mM ionic strength. The bulk concentration was here approximated with the number of molecules at 100 mM ionic strength. Applying expression (7) to the Rh6G values a surface potential of about −100 ± 9 mV at 0.1 mM ionic strength is deduced, which is similar to the cationic Rh123 values.

The reason for the slightly higher estimation for the potential might be the larger hydrophobicity of rhodamine 6G [29]. A comparison at 100 mM ionic strength give *N*_Rh6G_ = 1.48 and *N*_Rh123_ = 0.52, which is relatively well in agreement with the differences in concentrations. At 0.1 mM ionic strength the background corrected values are *N*_Rh6G_ = 5.93 compared to *N*_Rh123_ = 1.86, which gives a difference of about 3.2 for a sample concentration difference of only 2.5. The calculated strength of the surface potential is therefore probably biased in the case of rhodamine 6G. The inset of Figure 8 shows the axial passage-time, *τ_z_*, of rhodamine 6G, which indicates strong restricted motion with decreasing ionic strength. Again, a comparison to the passage time of rhodamine 123 reveals the same main trend, and where the small differences seen are probably due to different hydrophobicity of the two fluorophores. The difference is partly explained by Rh6G’s higher molecular mass, which contributes a factor of 1.08 to the difference of 1.4 seen in axial passages times at 100 mM ionic strength. Additional surface investigations with a combination of techniques (single particle tracking, confocal FCS, TIR-FCS, TIR-FM, *etc*.) are probably needed to disentangle the full complexity of this interfacial dynamics [9,26,27,29].

Switching from cationic fluorescent molecules (*q* = +1) to negatively charged fluorescein instead (anionic/dianionic, *q* = −1 to −2), allows probing contribution of repulsion at the glass interface [10,13]. Figure 9a shows background corrected number of fluorescein molecules in the vicinity of the surface extracted from TIR-FCS measurements (data not shown). Compared to the attraction of cationic dyes, the concentration is now decreasing as the ionic strength decreases. The deduced concentration ratio of molecules, *N*_x_, due to the repulsion is shown in the inset. As the Debye-length of the repulsion increases, the ratio decreases over two times, meaning that less than half the number of molecules is located within the electric double layer at low compared to high ionic strengths. In addition to the changes in number of molecules, the axial passage-time, *τ_z_*, and the normalized axial passage-times are also shown in Figure 9b. The results also give a clear indication of an electrostatic interaction repelling anionic/dianionic molecules from the negative glass surface. This repellent interaction forces fluorescein molecules to dwell mainly in the outer parts of the observation volume, resulting in a decreased axial passage-time at lower ionic strengths (*i.e.*, at a less screened surface).

Applying the fluorescein data to Equation (5) allows us to estimate the surface potential, which becomes about −13 ± 2 mV (pH = 6.6) at an ionic strength of 0.1 mM, when assuming a charge of *q* = −1.5. This mimics a mixture of the protonated and deprotonated form of fluorescein, HFl^−^ ←→ Fl^2−^ (pKa = 6.4) at this pH [22]. Assuming further that the protonated anionic form is practically nonfluorescent (meaning it is only the dianionic form that contributes to the TIR-FCS data), the surface potential then becomes about −10 ± 2 mV. Applying expression (7) to the fluorescein values (assuming *q* = 2) yields a surface potential of about −40 ± 4 mV at 0.1 mM ionic strength. The reason for the lower deduced surface potential might be due to a remaining electrostatic interactions even at 100 mM ionic strength, also seen by TIR-FM methods [10,11]. This decreases the number of molecules at the interface, and the ratio *N̄* / *N̄_B_*, leading to extraction of a lower surface potential *via* Equation (5). The bulk value should therefore have been measured at a higher ionic strength.

To circumvent the problems of bulk concentration estimates, a measurement of *N̄_B_* in a confocal FCS mode (with a known probe volume) could be done before switching to surface based TIR-FCS investigations. This approach has recently been applied to studies of the restricted motion of fluorescently labelled vesicles diffusing near planar membranes [28]. In that study it was also seen that the negatively charged probes experienced a repulsive force from negative surfaces. Deviation from theory was however seen at low ionic strengths, where the electrostatic decay lengths start to approach the size of the evanescent excitation profile. A pool of fluorescent probes might then be located outside the TIR-FCS detection volume, which leads to an underestimation of the measured concentrations, as pointed out above.

As a final example, neutral organic dyes (ATTO 488 and rhodamine 110, *q* = ±0) were also applied to probe the negative glass surface. ATTO 488 is uncharged while rhodamine 110 is zwitterionic and has at neutral pH an unprotonated carboxyl group carrying a negative charge and an amino group carrying a positive charge. Figure 10a shows the number of neutral probe molecules (corrected for background contributions) in the vicinity of the surface. As no main electrostatic charge-charge interactions are present, the measured concentration of both dyes stays constant for all ionic strengths. This is also true for the in Figure 10b shown axial passage-times. Neutral fluorescent molecules can therefore be used as calibration standards, as they basically show no electrostatic attraction or repulsion to charged surfaces. The small variations seen come from noise or measurement error. A closer look at the data for rhodamine 110 reveals, however, that the axial passage-time is somewhat higher than expected compared to ATTO 488, which has a higher molecular mass. The larger than expected values for the rhodamine 110 may reflect some hydrophobic interactions or very weak remaining electrostatic influences following from its zwitterionic character. Again, further studies are needed to investigate the full complexity of that interfacial interactions dynamics.

## 4. Conclusions

In this work we have investigated electrostatic interaction between negatively charged glass surfaces and several fluorophores of different charges (*q* = +1, ±0, −1, −2) used in ultrasensitive fluorescence microscopy. The interfacial dynamics were analyzed by correlation analysis in an objective-based total internal reflection fluorescence microscope (TIR-FCS). The dynamics of cationic rhodamine 123 and rhodamine 6G, anionic/dianionic fluorescein, zwitterionic rhodamine 110 and neutral ATTO 488 dyes was investigated at various ionic strengths at physiological pH. As analyzed by means of the amplitude and time-evolution of the autocorrelation function, the fluorescent molecules experienced electrostatic attraction or repulsion at the glass surface depending on their charges. Influences of the electrostatic interactions were also monitored through the triplet-state population and triplet relaxation time, including the amount of detected fluorescence or the count-per-molecule parameter. These TIR-FCS results provide an increased understanding of how fluorophores are influenced by the microenvironment of a glass surface, and show a promising approach for characterizing electrostatic interactions at interfaces. The importance to understand the responses of fluorescent probe molecules is fundamental when complex biological systems, such as, transport through membrane channels, or ligand binding to membrane receptor, are to be investigated [3]. As there is always a pool of free dyes present, which can interfere with the study, a good knowledge of the “interferers” interfacial response is crucial to disentangle the true biological questions. This method can serves as an excellent tool in investigating such dynamics as it may delivers multidimensional information content. It may also serve as a tool to compare different surface interaction models [43].
